# Toward the identification and role of structural variations during dog domestication

**DOI:** 10.1093/nsr/nwy086

**Published:** 2018-08-30

**Authors:** Christophe Hitte

**Affiliations:** Christophe Hitte University of Rennes, France Reviewer of NSR

To date, we know little about the genetic diversity and development mechanisms that, through the process of domestication, have made the modern dog emerge. Following the processes of domestication and a phase of intense artificial selection, dogs became our auxiliaries for hunting, guarding and finally our best friends. The range of phenotypic diversity in dogs today greatly exceeds that of its ancestors, the wolves, making dogs the most morphologically diverse species of terrestrial mammals [1]. To advance these domestication research questions, studies are now focusing on differences in the content, organization and structure of the genome, called structural variations (SV), as a major source of genetic and phenotypic diversity. In addition, SVs have also emerged to provide an important substrate for evolutionary innovations. SVs have marked genomic effects, altering gene expression, affecting gene dosing, losing regulatory elements, unmasking regulatory polymorphisms and affecting the evolution of new genes. In the article ‘Structure variation during dog domestication: insight from grey wolf and dhole genomes', Guo-Dong Wang *et al.* report a comprehensive analysis of the evolutionary dynamics of SVs in order to advance the understanding of domestication of dogs. The authors point out that most dog-domestication studies [2,3] have analysed SNPs and not yet SVs. However, the absence of large-scale analyses of the SVs of the dog genome does not make it possible to identify all the important genetic signals likely to clarify the domestication of the dog. The authors also note another drawback, namely the absence of genomic assemblages of ancestors and external groups of dogs—two essential elements for identifying ancestral and lineage-specific SVs, and facilitating the characterization of vital events in the evolutionary history of the dog species. The use of the dog genome [4] as a reference ignores information specific to the wolf, especially large chromosomal variations, and does not allow deciphering the evolutionary dynamics of the SV during domestication.

This work presents an annotated genome assembly of dog's wild ancestor, the grey wolf (*Canis lupus*), and the first *de novo* genome assembly of a dhole (*Cuon alpinus*) used as an outgroup. Through comparative analysis, this study identifies key dog-specific transposable elements (TEs), gene family alterations and SVs, and analyses their contribution to phenotypic variations that characterize dog domestication. The results of this work first demonstrate the importance of large-scale genomic variant analysis in domestication studies. The study provides a comprehensive landscape of SVs in dog genomes and assesses the significance of gene gains and losses during dog domestication, which are enriched in a variety of metabolic pathways and neurological processes. Of particular interest is the copy number gain of a carbohydrate metabolism gene, *AKR1B1*. This finding may imply that dogs tend to have up-regulated *de novo* fatty acid synthesis in the small intestine and liver compared to the grey wolf. This suggests that the high-starch diet during the agricultural revolution did influence carbohydrate metabolism [5] and also lipid synthesis and carbonyls detoxification in the domestic dog. Additional studies at the genome scale will be needed to further obtain results, including studies based on gene expression, that, with the advancement of high-throughput transcriptome sequencing, will increasingly illuminate the importance of coding and non-coding genes in evolutionary biology.
